# Chinese medicinal formula, Qinggan Huoxue Recipe protects rats from alcoholic liver disease via the lipopolysaccharide-Kupffer cell signal conduction pathway

**DOI:** 10.3892/etm.2014.1740

**Published:** 2014-05-28

**Authors:** TAO WU, TAO LIU, LI ZHANG, LIAN-JUN XING, PEI-YONG ZHENG, GUANG JI

**Affiliations:** 1 Research Center of Chinese Medicine Therapy and Systems Biology, Shanghai University of Traditional Chinese Medicine, Shanghai 201203, P.R. China; 2 Institute of Digestive Disease, Longhua Hospital, Shanghai University of Traditional Chinese Medicine, Shanghai 200032, P.R. China

**Keywords:** Qinggan Huoxue Recipe, alcoholic liver disease, lipopolysaccharide, Kupffer cells

## Abstract

The Chinese medicinal formula, Qinggan (QG) Huoxue (HX) Recipe (R) exerts a range of pharmacological effects, including reversible steatosis, decreased levels of inflammatory cytokines and lipid peroxidation resistance. The aim of the present study was to determine the specific mechanisms of QGHXR hepatoprotection through the lipopolysaccharide-Kupffer cell (LPS-KC) signal conduction pathway in rats with alcoholic liver disease (ALD). ALD rats were exposed to the compound factors, QGR and HXR. Hematoxylin and eosin staining was conducted to evaluate the pathological changes in the liver following QGHXR treatment and an enzyme-linked immunosorbent assay was performed to measure the content of tumor necrosis factor (TNF)-α in the plasma. Immunohistochemical staining was conducted to examine the expression of cell differentiation antigen (CD) 68 and 14. In addition, western blot analysis and reverse transcription-polymerase chain reaction were used to measure the expression of Toll-like receptor 4 (TLR_4_), phosphorylated-extracellular regulated protein kinases (p-ERK), nuclear factor (NF)-κB, CD14 and TNF-α. Following stimulation with the compound factors, the rats exhibited increased alanine aminotransferase (ALT) and aspartate aminotransferase (AST) levels, as well as marked pathological changes. Furthermore, the related molecules in the LPS-KC pathway were upregulated and QGHXR was identified to be effective in the LPS-KC signal conduction pathway in the ALD rats. QGHXR was superior to QGR and HXR in reducing the serum ALT and AST levels, regulating CD14, TLR_4_, NF-κB, ERK and TNF-α as well as improving the pathological changes. The results indicated that QGHXR therapy may provide a novel strategy for treating ALD via regulation of the related molecules in the LPS-KC signaling pathway.

## Introduction

Alcoholic liver disease (ALD) is a common clinical complication resulting from long-term alcohol abuse. Its morphological features include alcoholic fatty liver, hepatitis and cirrhosis. The pathogenesis of ALD is multifactorial and involves genetic, nutritional and environmental factors, in addition to numerous injurious factors, such as oxidative or nitrosative stress, bacterial lipopolysaccharide (LPS) and cytokines (1,2).

Alcohol-induced sensitization of liver macrophages by portal endotoxins/LPS is considered to be a hallmark of ALD. Intracellular mechanisms that are associated with LPS-induced signaling are critical in the initiation and progression of ALD. LPS recognition by Toll-like receptor 4 (TLR_4_) and cell differentiation antigen (CD14) on liver macrophages and activation of downstream signaling pathways, which culminate in the activation of transcription factors, such as nuclear factor (NF)-κB, lead to increased inflammatory cytokine production in ALD. In addition, LPS-induced mitogen-activated protein kinases (MAPKs), such as extracellular regulated protein kinases (ERKs) contribute to liver injury. The importance of alcohol-induced reactive oxygen species and interactions with TLR pathways in macrophages that lead to inflammation are becoming increasingly evident. Collectively, these signaling pathways induce pro- and anti-inflammatory cytokines, which are significant in ALD (3–5).

Qinggan (QG) Huoxue (HX) Recipe (R) exerts a broad range of pharmacological effects, including partially reversible steatosis, decreased fibrosis marker levels, decreased inflammatory cytokine levels and resistance to lipid peroxidation. The study group introduces the Yu-Re pathogenesis theory in ALD, which is based on clinical epidemiology and advocates the rule of QG and HX for the treatment of ALD (6). Yu-Re pathogenesis is based on our previous epidemiological investigation with 745 ALD patients. Shi-re and Yu-xue were the basic pathomechanism of ALD, therefore the rule of QG and HX were the basic therapy for ALD. The aim of the present study was to characterize the regulation of the LPS-Kupffer cell (KC) signal molecules by administering QGHXR to rats with ALD in order to determine the experimental basis for the use of traditional Chinese medicine in ALD therapy.

## Materials and methods

### Reagents

QGHXR (9 g bupleurum root, 9 g scutellaria root, 15 g red sage root, 9 g Carapax Trionycis and 15 g *Radix Puerariae*), QGR (9 g bupleurum root and 9 g *scutellaria* root) and HXR (15 g red sage root, 9 g Carapax Trionycis and 15 g *Radix Puerariae*), were concentrated to 4.75, 1.5 and 3.25 g/ml, respectively, and processed at the Department of Pharmacy, Longhua Hospital (Shanghai, China). In order to ensure the uniformity and stability of herbal products, we performed the HPLC method to detect the contents of four flavones including Puerarin, Baicalin, Baicalein and Wogonin (7). Rabbit polyclonal anti-phosphorylated (p)-ERK and mouse anti-rat NF-κB were purchased from Cell Signaling Technology, Inc. (Danvers, MA, USA) and anti-TLR_4_ was purchased from Santa Cruz Biotechnology, Inc., (Santa Cruz, CA, USA).

### Animal preparation

Specific pathogen-free male Wistar rats (n=100; weight, 180±10 g) were purchased from Slac Laboratory Animal Center, Inc. (Shanghai, China). Daily general observations and weekly body weights of the rats were recorded. The rats were randomly divided into three groups: The blank group (n=10), the carbon tetrachloride (CCl_4_) group (n=10) and the model group (n=80). The model group was administered with a 10 ml/kg/d dose mixture twice per day, which included 10 ml 60% alcohol, 2 ml corn oil and 25 mg pyrazole. Intraperitoneal injections of 0.25 ml/kg of 25% CCl_4_ in olive oil were administered twice a week from the second week (8); the CCl_4_ group only received intraperitoneal injections. Two rats from the model group were sacrificed each week for histological observation and after four weeks, the model group was divided into four subgroups: QGHXR (n=15), QGR (n=15), HXR (n=15) and the model group (the remaining rats). The animals were sacrificed with exsanguinating after blood samples were obtained from abdominal aorta. The model group was administered with 10 ml/kg saline per day, the QGHXR group received 200 mg/kg QGHXR, the QGR group received 137.5 mg/kg QGR and the HXR group received 62.5 mg/kg HXR. The blank and CCl_4_ groups were administered with saline and at the end of the six weeks, all of the rats were anesthetized with 2% pentobarbital sodium at the dosage of 2 ml/kg and sacrificed. Blood samples and liver tissue specimens were collected and a section of the liver was fixed for histopathology. Another section was used for polymerase chain reaction (PCR) and western blot analysis; the remaining tissue was stored at −80°C until the assays were conducted. All of the rats were handled according to the recommendations of the National Institutes of Health Guidelines for Care and Use of Laboratory Animals. The experimental protocol was approved by the Shanghai Medical Experimental Animal Care Committee (Shanghai, China).

### Serum alanine aminotransferase (ALT), aspartate aminotransferase (AST) and tumor necrosis factor (TNF)-α assays

Serum ALT and AST levels were assayed using a Hitachi 7170S Biochemical Analyzer (Hitachi Ltd., Tokyo, Japan). The serum TNF-α level was assayed using a mouse TNF-α enzyme-linked immunosorbent assay kit (Yusen Biotech Inc., Shanghai, China). A total of 50 μl standards (standard curve concentrations: 1,000, 500, 250, 125, 62.5, 32, 16 and 0 ng/ml) or serum was added to each well in duplicate, the absorbance was measured at 450–550 nm with Microplate Spectrophotometer (Bio-Tek Instruments Inc., Winooski, VT, USA) and the results were calculated using a standard curve.

### Pathology and immunohistochemistry

Small segments of liver tissue were fixed in 10% neutral buffered formalin and processed into paraffin sections for hematoxylin and eosin (H&E) or immunohistochemical staining. The morphological variations were observed by two pathologists who were blinded to the experimental information. Diehl *et al* (9) and Wang (10) have described the pathological changes in fatty degeneration, apoptosis and necrosis, which are observed by H&E staining, as well as the associated criteria. Immunohistochemical staining was performed with a streptavidin-biotin complex kit (Boster Biological Technologies, Inc., Wuhan, China) for CD14 using rabbit anti-CD14 antibodies (1:100; Boster Biological Technologies, Inc.) and CD68 with mouse anti-rat CD68 antibodies (1:200; Bio-Rad Laboratories, Hercules, CA, USA). The slides were visualized with 3,3′-diaminobenzidine and positive staining was indicated by a yellow-brown color. Evaluation of the specific positive reactions were performed using Image-Pro Plus 6.0 (Media Cybernetics, Inc., Rockville, MD, USA) and were presented as the integral optical density value.

### Semi-quantitative reverse transcription-PCR (RT-PCR) analysis for TNF-α, CD14 and TLR_4_

Total RNA was extracted from the liver tissue using the TRIzol reagent (Invitrogen Life Technologies, Carlsbad, CA, USA). The complementary DNA was synthesized from 2 μg total RNA using Moloney murine leukemia virus reverse transcriptase (Takara Co., Ltd., Japan) and mouse TNF-α, CD14 and TLR_4_ mRNA were amplified using the primers shown in Table I. The PCR analysis was conducted as follows: 26 Cycles at 95°C for 30 sec and at 60°C for 40 sec followed by a 1 min extension stage at 72°C. The amplification products were electrophoretically analyzed on 1.0% agarose gel containing 0.1 μg/ml ethidium bromide.

### Semi-quantitative western blot analysis for TLR_4_, p-ERK and NF-κB

Homogenates that contained equal quantities of the proteins (50 μg) were separated using SDS-PAGE and electrophoretically transferred onto a nitrocellulose membrane (Bio-Rad Laboratories). The nitrocellulose membrane was blocked overnight with 5% non-fat dry milk in phosphate-buffered saline with Tween-20 (0.1%, v/w) at 4°C. The membrane was incubated with the primary antibodies, which were diluted according to the manufacturer’s instructions for 16 h. Horseradish peroxidase-conjugated anti-rabbit or anti-mouse IgG (Santa Cruz Biotechnology, Inc.) served as secondary antibodies. The immunoreactive protein was visualized by ECL Photon Chemiluminescence Western Blotting Detection Kits (FIVEphoton Biochemicals, San Diego, CA, USA) and western blot analysis according to the manufacturer’s instructions.

### Replications and statistical analysis

Each result was independently replicated at least three times and certain results were repeated multiple times, when appropriate or required. All of the continuous variables were expressed as means ± standard deviation, and analysis of variance and post hoc tests were used to measure the statistical significance of differences compared with the control. The software package, SPSS 15.0 (SPSS Inc., Chicago, IL, USA) was used for the data analysis and P<0.05 was considered to indicate a statistically significant difference.

## Results

### Parameter observations in rat models

The rats became excited and ran around the cage following the administration of alcohol. Subsequently, they were unable to walk and eventually fell into a deep sleep. Furthermore, the weight of the rats noticeably reduced and the rats became cachectic. The daily diet, weight and energy of the rats in the QGHXR, QGR and HXR groups were better than those observed in the model group. The serum ALT and AST levels were upregulated to a greater extent in the model group compared with the control group; in addition, the related molecules in the LPS-KC pathway were identified to be partially activated in the model group. Twenty-one rats succumbed during the study; however, no animal experienced >10% QGHXR-related weight loss following the treatment regimen (data not shown).

### QGHXR reduces liver injury due to the reduction of ALT, AST and TNF-α levels in ALD rats

**Figure 1:**
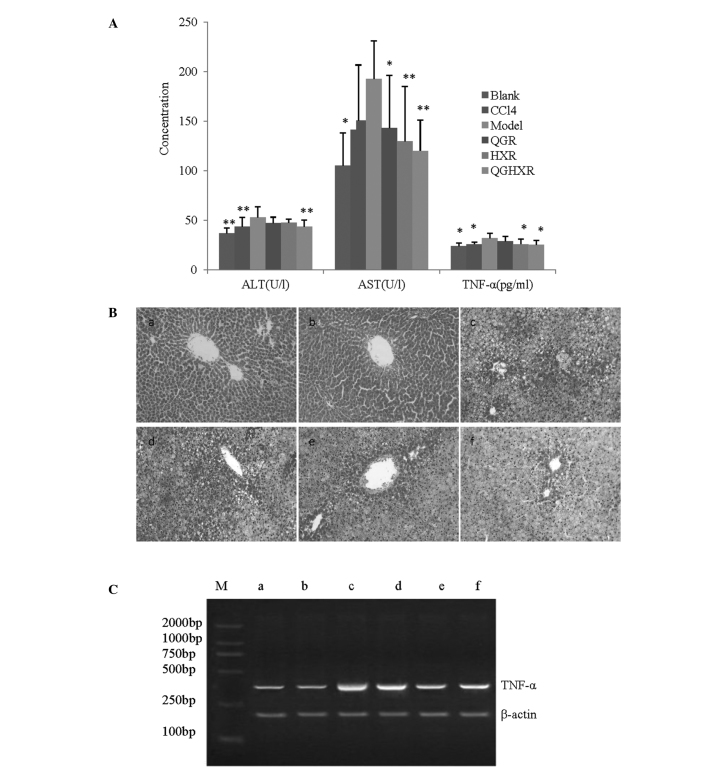
QGHXR improved liver injury by reducing ALT, AST and TNF-α levels in the ALD rats. (A) Serum ALT, AST and TNF-α levels of the ALD rats were significantly higher than those observed in the control group. Treatment with QGHXR decreased the ALT levels to a greater extent than QGR or HXR. Similar trends were observed in the AST and TNF-α levels. ^*^P<0.05 and ^**^P<0.01 compared with the model group. (B) Magnification, ×200. The liver in the model group (Bc) exhibited marker hepatic steatosis and inflammatory infiltrates of monocytes compared with the blank group (Ba). The CCl_4_ group (Bb) was identical to the blank group. The QGHXR treatment (Bf) significantly improved the pathological parameters compared with (Bd) QGR or (Be) HXR treatment. (C) The reverse transcription-polymerase chain reaction results revealed that QGHXR and HXR suppressed TNF-α mRNA expression; however, QGR showed no distinct effect on TNF-α mRNA. Lane M, marker; lane a, blank group; lane b, CCl_4_ group; lane c, model group; lane d, QGR group; lane e, HXR group; lane f, QGHXR group. CC1_4_, carbon tetrachloride; QGR, Qinggan Recipe; HXR, Huoxue Recipe; QGHXR, Qinggan Huoxue Recipe; ALT, alanine aminotransferase; AST, aspartate aminotransferase; TNF, tumor necrosis factor; ALD, alcoholic liver disease; mRNA, messenger RNA.

QGHXR reduced the serum ALT, AST and TNF-α levels by 17.98% (43.53±6.66 vs. 53.07±10.65 U/l), 37.65% (120.09±31.15 vs. 192.62±38.43 U/l) and 21.86% (24.99±4.65 vs. 31.98±4.61 pg/ml), respectively, compared with the model group. QGR significantly decreased the AST levels, however, it did not affect ALT and TNF-α. HXR reduced the AST and TNF-α levels without affecting the ALT levels (Fig. 1A). The liver samples from the model group presented with hepatic steatosis and inflammatory monocytic infiltrates when compared with the samples from the blank group (Fig. 1Ba and b) and the CCl_4_ group appeared to be identical to the blank group (Fig. 1Bc). The QGHXR treatment significantly improved the aforementioned pathological changes compared with the QGR or HXR treatments (Fig. 1Bd–f). Furthermore, the RT-PCR results identified that QGHXR and HXR suppressed TNF-α mRNA expression, however, no distinct QGR effect was evident on TNF-α mRNA (Fig. 1C).

### QGHXR downregulates CD68, CD14 and TLR_4_ expression in ALD rat liver tissue

**Figure 2:**
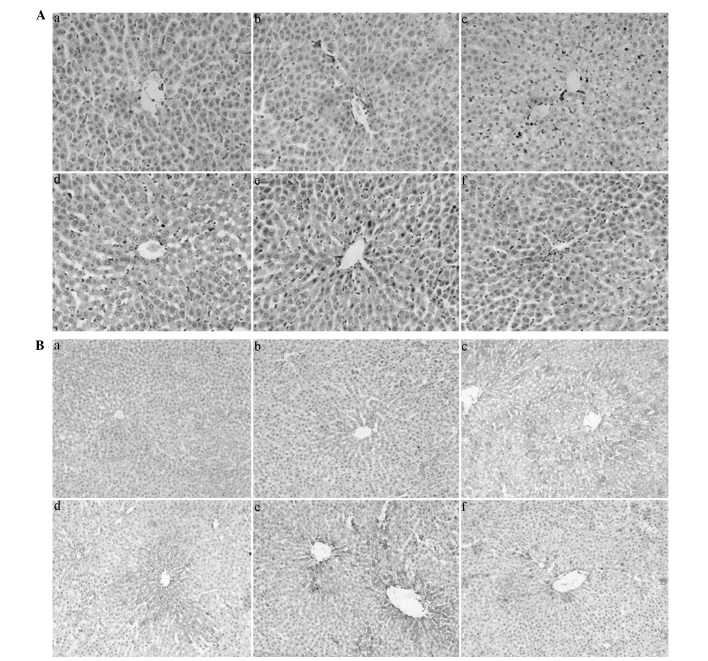
QGHXR downregulated CD68 and CD14 expression in the liver tissue of the ALD rats. (A) Magnification, ×400. (Aa) Marginal CD68 positive staining was observed in the sinus hepaticus, the portal area of the hepatic lobules and in the liver of the blank group. (Ab) No obvious change in the CCl_4_ group was evident. (Ac) Evident CD68-positive staining was observed in the liver of the model group, concentrating in the sinus hepaticus where steatosis and inflammatory cell infiltrates were apparent. (Af) QGHXR and (Ae) HXR inhibited Kupffer cell activation; however, (Ad) QGR did not show any significant effect. (B) Magnification, ×200. (Ba) Immunohistochemistry showed a small positively stained area in the cytoplasm of the liver from the blank group, which was predominantly situated on the sinus hepaticus or non-parenchymal cells around the central veins. (Bb) No obvious change in the CCl_4_ group was evident. (Bc) An obvious CD14-positive area was observed in the model group. (Bf) QGHXR decreased the CD14 expression in the model rats; however, (Bd) QGR and (Be) HXR did not significantly effect the CD14 expression. QGHXR, Qinggan Huoxue Recipe; CD, cell differentiation antigen; ALD, alcoholic liver disease; CC1_4_, carbon tetrachloride; HXR, Huoxue Recipe; QGR, Qinggan Recipe.

**Figure 3:**
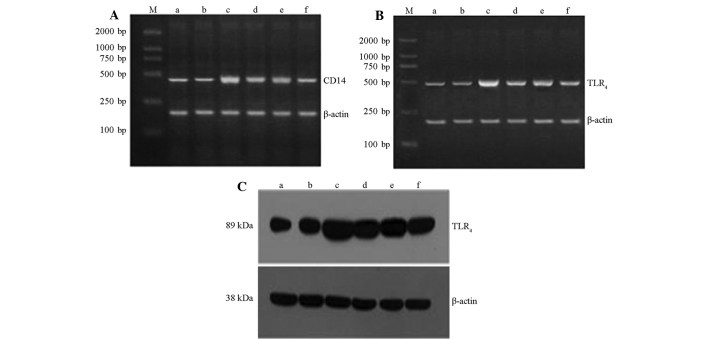
QGHXR downregulated CD14 and TLR4 expression in the liver tissue of the ALD rats. Reverse transcription-polymerase chain reaction results for (A) CD14 and (B) TLR4. (C) Western blot analysis results for TLR4. Lane M, marker; lane a, blank group; lane b, CCl_4_ group; lane c, model group; lane d, QGR group; lane e, HXR group; lane f, QGHXR group. QGHXR, Qinggan Huoxue Recipe; CD, cell differentiation antigen; TLR4_,_ Toll-like receptor 4; ALD, alcoholic liver disease; CC1_4_, carbon tetrachloride; QGR, Qinggan Recipe; HXR, Huoxue Recipe.

QGHXR and HXR inhibited KC activation (Fig. 2Aa–f) and immunohistochemistry and western blot analysis demonstrated that QGHXR decreased the CD14 and TLR_4_ protein levels in the cytoplasm of the liver (Figs. 2Ba–f and 3C). However, QGR and HXR showed no effect on CD14 and TLR_4_ protein levels. In addition, the RT-PCR results showed that the QGHXR treatment decreased the overexpression of CD14 and TLR_4_ mRNA, as well as identifying that QGHXR was more effective than HXR and QGR (Fig. 3A and B).

### QGHXR inhibits hepatic ERK activation and NF-κB expression

**Figure 4:**
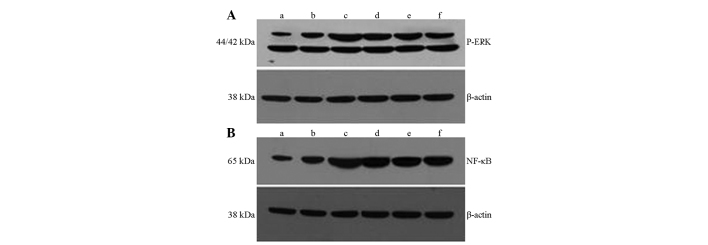
QGHXR inhibited hepatic ERK activation and NF-κB expression in the liver tissue of the ALD rats. Western blot analysis results for (A) p-ERK and (B) NF-κB. Lane M, marker; lane a, blank group; lane b, cell differentiation antigen 14 group; lane c, model group; lane d, Qinggan Recipe group; lane e, Huoxue Recipe group; lane f, QGHXR group. p-ERK, phosphorylated-extracellular regulated protein kinase; NF, nuclear factor; QGHXR, Qinggan Huoxue Recipe.

The expression of hepatic p-ERK and NF-κB in the ALD rats was upregulated compared with that of the normal and CCl_4_ group (Fig. 4A and B). ERK activation and NF-κB expression were inhibited in the QGHXR group and no significant changes in p-ERK expression were apparent. QGHXR showed greater inhibition of NF-κB expression when compared with QGR.

## Discussion

There is increasing evidence indicating that Chinese medicinal formulas may be adopted as therapeutic agents or adjuvants in ALD treatment (6,11). In the present study, a Wistar rat model, which has previously been successfully adopted for the screening of therapeutic agents for ALD (7), was used and QGHXR was demonstrated to exert a range of pharmacological effects for improving liver injury without extensive toxicity. The rule of QG and HX were the basic therapy for ALD. QGHXR exerts effects on treating ALD. Therefore we separated the whole formulation QGHXR into QGR and HXR in order to determine their roles in ALD. In the present study, a 0.25ml/kg of 25% CCl_4_ served as the coefficient of alcohol as it reduces the model cycle time and enhances its stability (6). The CCl_4_ group was identical to the blank group except for the marginally increased AST level (data not shown), which indicated that alcohol is the predominant cause of liver injury.

Chronic ethanol consumption leads to the elevation of hepatic LPS levels that target CD14/TLR_4_ receptors, which are also elevated following ethanol consumption, leading to the production of pro-inflammatory cytokines, such as TNF-α, IL-6 and transforming growth factor-β1, and the subsequent activation of hepatic stellate cells (HSCs) and liver fibrosis (12,13). The following studies were conducted to investigate the underlying mechanism of the effect of QGHXR on ALD.

The liver is composed of parenchymal cells, such as hepatocytes and non-parenchymal cells, such as sinusoidal endothelial cells, KCs, HSCs, dendritic cells and other lymphocytes. Previous studies indicated that KCs were predominantly involved in alcohol-mediated inflammation via LPS/TLR_4_ signaling-dependent mechanisms (14,15) in addition to indicating that QGHXR and HXR inhibit KC activation.

The currently accepted model of ALD indicates that LPS promotes hepatic injury via the induction of KC activation, resulting in the production of TNF-α and additional inflammatory mediators (16,17). Significant evidence of the pivotal role of TNF-α in alcohol-induced liver injury was identified through a study, which used anti-TNF-α antibodies to prevent liver injury in alcohol-fed rats (18) as well as the observation that mice lacking a TNF type I receptor did not develop ALD (19,20). QGHXR and HXR significantly reduced TNF-α mRNA, however, QGR demonstrated no distinct effect; the TNF-α protein expression was similar to that of TNF-α mRNA. QGHXR and HXR protected the hepatocytes by reducing TNF-α generation and QGR showed no significant effect on the TNF-α gene and protein regulation, which was consistent with the results relating to the regulation of KC activation.

Furthermore, immunohistochemistry and western blot analysis indicated that QGHXR decreased CD14 expression in the cytoplasm, but not QGR or HXR. CD14 is a glycosylphosphatidylinositol-anchored protein that also exists in a soluble form. It facilitates the transfer of LPS to the TLR_4_/lymphocyte antigen 96 (MD2) receptor complex and modulates LPS recognition (21,22). A recent study demonstrated that the expression of cytokines, such as TNF-α were inhibited following the addition of CD14 monoclonal antibodies (23). In addition, sensitivity to LPS increased 100–1,000 times following CD14 cDNA transfection into low endotoxin-reactive cells that do not express CD14 (24). Therefore, QGHXR decreased the efficiency of endotoxin signal conduction, and reduced the production of inflammatory factors and hepatocellular injury by regulating CD14 expression.

TLR_4_ recognizes the LPS lipid A motif, a suggested cofactor in the pathogenesis of ALD (25). Moreover, TLR_4_ is a predominant component of the LPS recognition receptor complex, which involves the co-receptors CD14, MD2 and the LPS binding protein (LBP) (26). Studies in knockout mouse models have shown that chronic alcohol feeding in CD14-, TLR_4_- and LBP-deficient mice resulted in the alleviation of alcohol-induced liver injury, indicating the significance of the TLR_4_ pathway (27–29). QGR, HXR and QGHXR significantly decreased TLR_4_; however, QGHXR exhibited a superior effect compared with HXR and QGR, which indicated that QGHXR decreased TLR_4_ expression and inhibited further signal transmission.

Changes in related molecules, such as p-ERK and NF-κB, were measured to determine the influence of QGHXR on the LPS-KC pathway. NF-κB is a central regulator of cellular stress in all liver cell types, forms p65/p50 heterodimers in macrophages and binds to the promoter region of various pro-inflammatory genes, which results in gene transactivation (30). The hepatic macrophage expression of pro-inflammatory mediators is predominantly regulated by NF-κB and murine models of chronic alcohol administration demonstrated increased NF-κB DNA binding in the liver (31). Chronic alcohol intake is hypothesized to prime the liver via sustained NF-κB activation and induction of basal and LPS-stimulated TNF-α. LPS recognition activates the MAPK family members, including ERK1/2, p38 and c-Jun N-terminal kinase, resulting in TNF-α production (32). In addition, chronic alcohol intake activates LPS-induced ERK1/2 activation, which contributes to TNF-α expression in murine hepatic macrophages (33). Recently Karki *et al* found that extract of buckwheat sprouts inhibited pro-inflammatory mediators IL-6 and TNF-α production in lipopolysaccharide-stimulated macrophages (RAW264.7) (34). Nwozo *et al* evaluated the protective effects of oils from *Zingiber officinale* (ginger) and *Curcuma long*a (turmeric) on acute ethanol-induced fatty liver in male Wistar rats (35). The results of the present study showed that QGHXR and its separate components, HXR and QGR, significantly decreased p-ERK and NF-κB expression, thus indicating that the therapeutic effect of QGHXR on ALD rats may be due to p-ERK and NF-κB downregulation.

In the present study, QGHXR was indicated to be a potent sensitizer for ALD in experimental rats. QGHXR regulated the membrane receptor, protein kinase, NF and abnormal function of the cytokine network via the LPS-KC pathway. Chinese herbal medicine appears to manifest its activity slowly, therefore, improving the agent administration strategy due to an earlier administration time and a longer therapeutic period, which may enhance the performance of the agent.

There were certain limitations in the present study; the exact mechanism of QGHXR protection against ALD was not identified; thus, further investigation into the stimulating effect is required. As a result of using rats, the ability to obtain robust evidence was limited, therefore, large-scale multicentric placebo-controlled prospective studies are required to verify the results. Regardless of these limitations, the present study provided preliminary data to support future QGHXR evaluations. Through observation of the multi-element, multichannel and multitarget action characteristics of Chinese medicine, QGHXR may be screened and the formulations simplified to establish the foundation for identifying their composition and active components.

In conclusion, the Chinese medicinal formula, QGHXR, is a potential treatment for ALD. The present study provided further clarification of the mechanism for QGHXR as a treatment for ALD via the LPS-KC pathway. Although the underlying mechanisms that govern these effects remain undetermined, the available evidence collectively demonstrated that QGHXR may be of therapeutic benefit in a clinical setting, indicating its potential use as an agent for protecting against ALD.

## Figures and Tables

**Table I:** Sequence-specific primers of TNF-α, CD14, TLR_4_ and β-actin.

Gene	Product (bp)	Forward	Reverse
TNF-α	379	5′-ATCGGTCCCAACAAGGAGGAGAAGT-3′	5′-TCCTTAGGGCAAGGGCTCTTGATGG-3′
CD14	481	5′-TCGGCTTGTTGCTGTTGCCTTTGAC-3′	5′-TTCTGCGAGCCAGGTATCCGTTGTT-3′
TLR4	500	5′-GGATTTTACGAATTCCACCTGTTAT-3′	5′-CGATACAATTCGACCTGCTGCCTCA-3′
β-actin	224	5′-TGTGATGGTGGGTATGGGTCAGAAG-3′	5′-TCACGGTTGGCCTTAGGGTTCAGAG-3′

TNF-α, tumor necrosis factor-α; CD14, cell differentiation antigen 14; TLR4, toll-like receptor 4.
